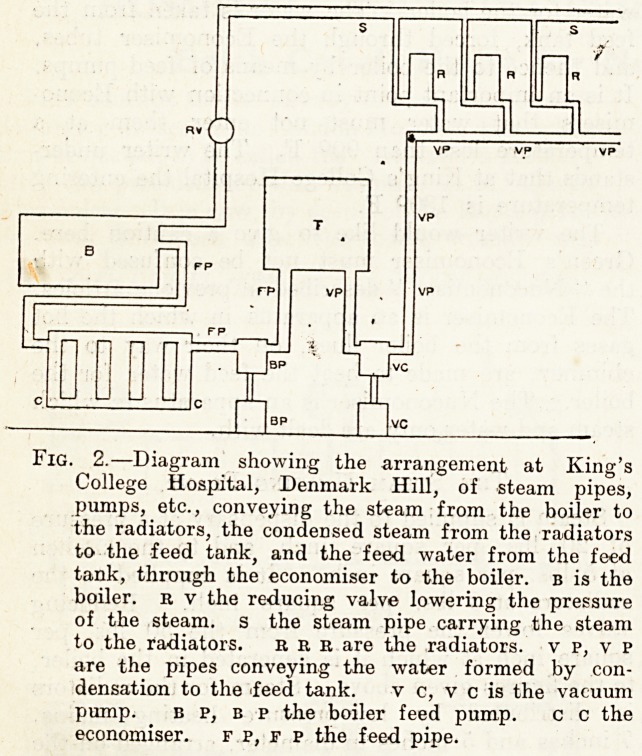# The New King's College Hospital at Denmark Hill

**Published:** 1916-01-15

**Authors:** 


### THE HEATING OF HOSPITALS.*

NOTE.—In a series of articles the writer proposes to describe the different systems of heating that are employed in hospitals. He will be pleased to answer any questions through "The Hospital," bearing upon the subject of heating or ventilation. Each system will be illustrated and described as exemplified at some hospital where it has been applied.

### VI. The New King's College Hospital at Denmark Hill.

### Heating by Open Grates Assisted by Low-Pressure Steam Radiators.

There are two or three important points in connection with the heating arrangements at the New King's College Hospital. Thus electricity for lighting and power is generated on the ground, but though steam is employed for heating, the dynamos are driven by Diesel engines, and the water that is heated by passing through the jackets of the engines is taken to the calorifiers, and heated to a higher temperature for the domestic hot-water supply. Diesel engines require that water shall be kept circulating through jackets outside of their cylinders, or they will not continue to work satisfactorily. The heating of the wards is by open-hearth stoves placed in the middle of the floor towards each end of the ward, as shown in the illustration, fig. 1. The stoves have two fire-

Fig. 1.—The Open-hearth Stove at King's College Hospital.

places each, as shown. Inlet flues, bringing fresh air to the stoves, and outlet flues, carrying off the hot gases, smoke, etc., are fixed under the floors. Steam-heated radiators are used to supplement the fireplaces, particularly during cold weather. Provision has been made for increasing the number of radiators should necessity arise. In the laboratories and parts of the basement, pipes are fixed near the ceiling in place of radiators.

### The Boiler Plant.

The boiler plant consists of three Lancashire boilers each 30 feet long by 8 feet in diameter. They are constructed to work up to a pressure of 80 lbs. per square inch if required. At present they are worked at 50 lbs. Anthracite coal is burnt in the boiler furnaces, and they are worked by forced draft, the air being heated on its way to the furnaces by passing over a nest of pipes through which the exhaust gases from the Diesel engine flow. With all internal combustion engines, about 30 per cent. of the heat liberated in the cylinder of the engine, on explosion, is wasted in the exhaust gases.

At King's College Hospital a portion is recovered by heating the air for the boiler. The temperature of the air is raised by about 20° F. above that of the surrounding atmosphere. The air is forced through the chamber containing the pipes in which the exhaust gases are flowing by a fan driven by an electric motor. Provision is made also, in case of failure of the exhaust gases from the Diesel engines, for warming the air by forcing it over a grid of steam-pipes.

There is a Green's Economiser, consisting of the usual 120 tubes, employed for heating the feed water for the boiler. The water is taken from the feed tank, forced through the Economiser tubes, and thence to the boiler by means of feed pumps. It is an important point in connection with Economisers that water must not enter them at a temperature less than 90° F. The writer understands that at King's College Hospital the entering temperature is 180° F.

The writer would like to give a caution here. Green's Economiser must not be confused with the "Nuconomiser" described in previous articles. The Economiser is an apparatus in which the hot gases from the boiler flues, on their way to the chimney, are made to heat the feed water for the boiler. The Nuconomiser is an apparatus in which steam and water only are dealt with.

### The Steam Heating Plant.

Steam is supplied to the dispensary at a pressure of 20 lbs. per square inch, and to the kitchen at 5 lbs. per square inch. It is supplied to the radiators at 3 lbs. per square inch. Reducing valves lower the pressure from the 50 lbs. per square inch at which it is generated in the boiler, to the figures given above. Steam for the radiators is distributed by low-pressure heating mains, 7 inches and 5 inches in diameter, arranged on the tree system. Exhaust mains run practically side by side with the steam supply mains, and vacuum pumps, at the central heating station, exhaust all of the steam from the radiators, and from the kitchen, except from the vegetable cookers, and deliver it to the feed tank. The feed tank acts as a condenser, and it also answers to the hot well employed in some heating installations. There is a certain amount of loss of water due to the conversion from water to steam and back to water, and in the passage of the steam from the boiler through the different parts of the apparatus. This loss is made up by water taken from the tanks on the roof of the hospital, the

* Previous articles appeared on July 31, September 11, October 2 and 16, and November 13, 1915.

cold "make-up" feed water, as it is called, condensing the steam coming from the radiators, kitchens, etc., while the temperature of the make-up is itself raised to that at which it can safely be pumped into the Economiser. Fig. 2 is a diagram showing the course of the steam from the boiler through the various apparatus back to the boiler again. The radiators are of the "Hospital" hinged type, arranged to swing out from the wall so that cleaning can easily be carried out. Each radiator is fitted with a hand regulating valve and a thermostatic valve, the latter controlling the flow of steam through the radiator, and being itself controlled by a thermostat in the ward. Ventilators are fitted behind the radiators in the walls, and are arranged so as to be easily cleaned. The kitchens are ventilated by exhaust fans fixed near the ceiling and delivering the vitiated air above the level of the hospital.

Heat is required in the hot serving closets, the linen rooms, the patients' clothes rooms, etc., in summer when the steam-heating system is not in use; to meet these requirements, hot-water radiators, pipes, etc., are fixed, supplied from the domestic hot-water service.

Fig. 2.—Diagram showing the arrangement at King's College Hospital, Denmark Hill, of steam pipes, pumps, etc., conveying the steam from the boiler to the radiators, the condensed steam from the radiators to the feed tank, and the feed water from the feed tank, through the economiser to the boiler. B is the boiler. R V the reducing valve lowering the pressure of the steam. S the steam pipe carrying the steam to the radiators. R R R are the radiators. V P, V P are the pipes conveying the water formed by condensation to the feed tank. V C, V C is the vacuum pump. B P, B P the boiler feed pump. C C the economiser. F P, F P the feed pipe.

### The Hot-water Supply.

The water for domestic requirements, baths, etc., is heated by a battery of five storage calorifiers, each holding 850 gallons of hot water, and each capable of providing 3,000 gallons of water per hour at a temperature of 180° F. to 200° F. The hot water from the jackets of the Diesel engine is used as far as it will go, the remaining water required being obtained from the tanks on the roof. The steam for the calorifiers is reduced to 20 lbs. per square inch. The quantity of steam entering each calorifier is controlled automatically by an American apparatus called the "Ideal Sylphon Tank Regulator." It consists of a small metallic bellows filled with a volatile liquid, and connected to the water space of the calorifier by a flexible copper pipe. The end of the flexible pipe is placed inside a larger tube, held inside the calorifier, so that a certain length of the flexible tube is exposed to the temperature of the hot water. The metallic bellows controls the valve through which steam enters the calorifier, and opens or closes the valve as the temperature of the water falls or rises. It is claimed that it maintains the temperature of the water within 2° F. on each side of the figure at which it is set. If water is required at 180° F. for instance, the Sylphon Regulator keeps it between 178° F. and 182° F.

The hot-water service is arranged on the thermo-syphon system, with an accelerator driven by an electric motor to increase the rate of flow.

### The Use of Electricity for Heating.

The committee of the hospital carefully considered the question of heating by electricity when the designs were being prepared. It was thought, however, that at present electric heating was not sufficiently advanced to warrant its adoption. It is employed on a small scale in the duty rooms of the wards, for making tea and boiling eggs. A cylindrical vessel filled from the main-water system is heated by internal coils connected with the hot-water supply main, so that the water in the cylinder is always at a certain temperature. For making tea and for boiling eggs, electrically heated kettles, egg-boilers, etc., take their water from this cylinder, so that the quantity of electricity required to bring them to the boiling-point, and in the case of the eggs to keep the water boiling for the time required, is very trifling, but the convenience is great.

The writer understands that the cost of generating electricity by means of the Diesel engines is 0.77d. per Board of Trade unit. For boiling water that is kept at a comparatively high temperature in the manner described, the quantity of electricity required would be small, so that the cost should be only a small fraction of a penny. The cost of generating electricity given above included fuel, wages, repairs, and accessories, but did not include standing charges or supervision. For plant of that size the figures are very favourable.

### The Steam and Hot-water Pipes.

The whole of the pipes are carried in ducts below the ground-floor passages, which, wherever it has been possible, have been made large enough for the attendant to walk from end to end. In certain portions this has not been possible, and removable covers are provided at frequent intervals so that access can be obtained to any portion of the pipes. The pipes leading to the upper storeys have been carried on the outside of the walls, covered with non-conducting materials, and the entry pipes to the wards are concealed in ingenious ways. The heating of the operation theatre claims separate notice later. The heating plant was installed by Messrs. Slater & Co., of Wells Street, London, W.

## Figures and Tables

**Fig. 1. f1:**
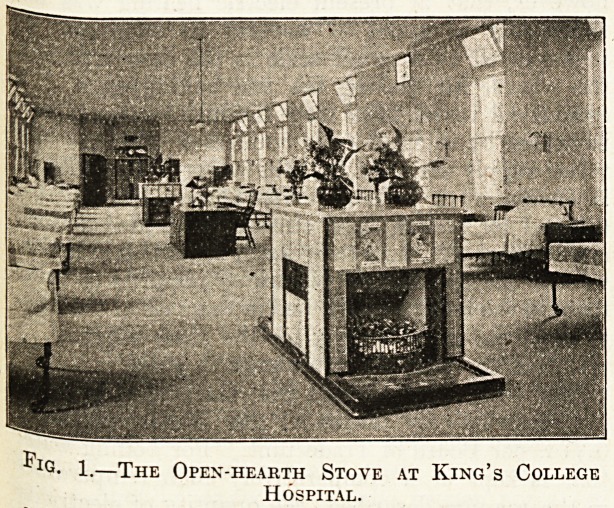


**Fig. 2. f2:**